# An Sp1/Sp3 Binding Polymorphism Confers Methylation Protection

**DOI:** 10.1371/journal.pgen.1000162

**Published:** 2008-08-22

**Authors:** Yanis A. Boumber, Yutaka Kondo, Xuqi Chen, Lanlan Shen, Yi Guo, Carmen Tellez, Marcos R. H. Estécio, Saira Ahmed, Jean-Pierre J. Issa

**Affiliations:** 1 Department of Leukemia, M. D. Anderson Cancer Center, University of Texas, Houston, Texas, United States of America; 2 Program in Cancer Biology, Graduate School of Biomedical Sciences, University of Texas, Houston, Texas, United States of America; Massachusetts General Hospital, Howard Hughes Medical Institute, United States of America

## Abstract

Hundreds of genes show aberrant DNA hypermethylation in cancer, yet little is known about the causes of this hypermethylation. We identified RIL as a frequent methylation target in cancer. In search for factors that influence RIL hypermethylation, we found a 12-bp polymorphic sequence around its transcription start site that creates a long allele. Pyrosequencing of homozygous tumors revealed a 2.1-fold higher methylation for the short alleles (P<0.001). Bisulfite sequencing of cancers heterozygous for RIL showed that the short alleles are 3.1-fold more methylated than the long (P<0.001). The comparison of expression levels between unmethylated long and short EBV-transformed cell lines showed no difference in expression *in vivo*. Electrophorectic mobility shift assay showed that the inserted region of the long allele binds Sp1 and Sp3 transcription factors, a binding that is absent in the short allele. Transient transfection of RIL allele-specific transgenes showed no effects of the additional Sp1 site on transcription early on. However, stable transfection of methylation-seeded constructs showed gradually decreasing transcription levels from the short allele with eventual spreading of *de novo* methylation. In contrast, the long allele showed stable levels of expression over time as measured by luciferase and ∼2–3-fold lower levels of methylation by bisulfite sequencing (P<0.001), suggesting that the polymorphic Sp1 site protects against time-dependent silencing. Our finding demonstrates that, in some genes, hypermethylation in cancer is dictated by protein-DNA interactions at the promoters and provides a novel mechanism by which genetic polymorphisms can influence an epigenetic state.

## Author Summary

The factors that guide DNA hypermethylation in cancer are poorly understood. We identified the candidate tumor-suppressor gene, RIL, as a frequent methylation target in cancer. Here, we report on a 12-bp polymorphic sequence around its transcription start site that creates a long allele. Methylation analysis showed that, in aging colon, colon cancer, and leukemias, the short allele had 2.1–3.1-fold higher methylation than the long allele (P<0.001). Short and long alleles had similar expression levels in EBV-transformed cell lines. Electrophorectic mobility shift assay showed that the inserted region of the long allele binds Sp1 and Sp3 transcription factors. Transfection of RIL allele-specific transgenes showed no effects of the additional Sp1 site on transcription early on, but methylation-seeded constructs showed gradually decreasing transcription from the short allele with eventual spreading of *de novo* methylation. By contrast, the long allele showed stable expression over time as measured by luciferase, and ∼2–3-fold lower levels of methylation by bisulfite sequencing (P<0.001), suggesting that the polymorphic Sp1 site protects against time-dependent silencing. Our finding demonstrates that in some genes, hypermethylation in cancer is dictated by protein-DNA interactions at the promoters and provides a novel mechanism by which genetic polymorphisms can influence an epigenetic state.

## Introduction

Causes of promoter DNA hypermethylation in cancer are unknown. Possibilities range from random events selected for to a model whereby an initial drop of transcription rate allows elimination of active chromatin boundaries and spreading of DNA methylation from “DNA methylation centers”- perhaps, repeat elements [1]. Alternatively, aberrant methylation might be caused by a repressor binding to the promoter and altering chromatin state to a closed configuration, which eventually causes abnormal methylation via recruitment of DNA methyltransferase [2],[3]. Furthermore, methylation seeding and ongoing transcription as well as possibly transcription factor binding seem to be required for the protection of promoters against methylation [4]. Nevertheless, the details and order of events in these processes remain elusive.

RIL is a ubiquitously expressed gene, which was originally identified as a candidate tumor suppressor [5]. Human RIL maps to chromosome 5q31.1, a region frequently deleted in the malignant cells of patients with myelodysplastic syndrome (MDS) and acute myelogenous leukemia (AML) [6], and appears to be a good candidate for one of the tumor-suppressor genes that reside in this area. Using methylated CpG island amplification [7] in the K562 cell line, we identified RIL as a novel gene aberrantly methylated in cancer. RIL CpG island is unmethylated in normal tissues, and is one of the most frequent targets for hypermethylation in various cancer cell lines and primary tumors. Hypermethylation of RIL correlates with loss of gene expression, which could be restored in methylated cell lines by 5-aza-dC. Moreover, RIL reexpression leads to a suppression of tumor cell growth and clonogenicity in soft agar as well as sensitization of cells to apoptosis [8].

Here, we describe a polymorphism in the RIL promoter that creates an Sp1/Sp3 binding site and protects against methylation in cancer.

## Results

**Figure 1:**
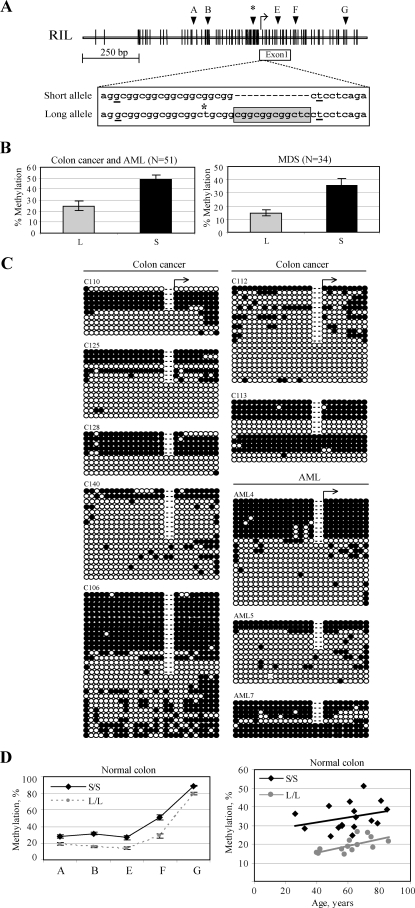
Identification of RIL Promoter Polymorphism and Preferential Methylation of the Short Allele in Cancer. (A) Map of RIL the CpG island. The distribution of CpG sites is represented by vertical bars. The transcription start site is indicated by an arrow. Letters A to G, location of pyrosequencing assays. Asterisk above the CpG map indicates the location of the COBRA assay. RIL exon 1 is shown as a box below the CpG island. The inserted 12-bp region in the long allele within the untranslated region of exon1 is shown in a grey box, with the upstream t is changed to g (t marked with an asterisk). Two previously described transcription start sites are underlined. (B) RIL shows lower methylation in homozygous long samples. *Left*, graphic representation of average RIL methylation values+/−SEM determined by pyrosequencing with assay A in 18 long (L/L) and 30 short (S/S) primary colon and AML samples are shown. *Right*, graphic representation of average RIL methylation values+/−SEM determined by pyrosequencing with assay B in 11 long (L/L) and 23 short (S/S) primary MDS samples are shown. (C) Heterozygous cases (L/S) have preferential methylation of RIL short allele. Bisulfite sequencing results are shown for the heterozygous colon tumors C110, C125, C128 and C140, C106, C112, C113; and heterozygous AML cases AML4, AML5, AML7 (*right*). Open and closed circles represent unmethylated and methylated CpG dinucleotides, respectively. Each line of circles represents a single cloned allele. The deletion region is shown by a dashed line. The proximal transcription start site is indicated by an arrow. Overall, there is a 3.1-fold difference (P<0.001) in the methylation status of RIL long and short alleles (especially obvious in C110, C125, C128; AML4, AML5). (D) *left*, average methylation values+/−SEM of 17 homozygous short (black line) and 14 homozygous long (grey dashed line) normal colon samples measured using pyrosequencing assays A to G; *right*, correlation between average methylation values and age for the assays A to F among 17 short (black diamonds) and 14 long (grey circles) normal colon samples.

In search for causes of aberrant hypermethylation of RIL in cancer, a ∼500-bp region around the transcription start site of the RIL promoter was sequenced, and a previously unknown polymorphic insertion was identified in the gene. In the population, 2 alleles of RIL exist, long and short, with a polymorphic area near a CGG repeat sequence adjacent to the transcription start site. The long allele is created by insertion of a 12-bp fragment (CGGCGGCGGCTC) and a substitution of T to G 3 bases upstream of the insertion site in the short allele (Figure 1A). *In silico* analysis identified additional putative binding sites for 3 transcription factors which appear only in the long allele. To more fully characterize this phenomenon and determine the frequency of alleles, a total of 326 normal samples were sequenced, which included 227 normal colon and 99 normal blood samples. In the normal population, 45% of people are homozygous for the short allele, 44% heterozygous and 11% homozygous for the long RIL allele. We have also determined the frequency of the RIL alleles among 240 cancer specimen which included 113 colon cancers, 100 MDS and 27 AML samples. We found that 44% of those were homozygous for the short allele, 40% heterozygous and 16% homozygous for the long RIL allele.

Differential methylation of the two RIL alleles was initially discovered after development of a COBRA assay. We initially noticed that methylated heterozygous tumors analyzed by COBRA seem to have preferential methylation of the short allele. After digestion of the COBRA products obtained from several homozygous and heterozygous tumors, we observed that among the 2 allele-specific bands, the predominant one is the lower band, which represents methylated short allele molecules (Figure S1). This led us to a suspicion that the short allele is more methylated than the long. For further studies, we decided to use pyrosequencing method, which is more quantitative in detecting methylation. To make sure that no amplification bias exists in detecting methylation, we designed all the assays primers either upstream or downstream of the insertion. We then tested the pyrosequencing assay A (Figure 1A) by studying mixed normal unmethylated with methylated DNA at known ratios, and found no bias in amplifying unmethylated vs. methylated DNA (Figure S2). Next, we studied methylation in 48 primary cancer samples (18 homozygous long and 30 homozygous short) which included 44 colon cancer and 4 AML cases with this assay, and found that homozygous short tumors showed mean 51.7%+/−3.8% methylation density vs. mean 24.7%+/−4.5% methylation for homozygous RIL long tumors, and thus had a 2.1 fold increased methylation density (P<0.001) (Figure 1B). We also studied 34 MDS samples (which included 11 homozygous long and 23 homozygous short) using pyrosequencing assay B (see Figure 1B) and found similar results: homozygous short RIL samples showed mean 35.5%+/−5.4% methylation density vs. mean 14.7%+/−2.4% methylation for homozygous RIL long samples, P = 0.001) (a 2.4 fold difference). To confirm the latter observations, we performed bisulfite sequencing of 10 methylated tumors with heterozygous alleles (6 colon cancer and 4 AML). These data showed that in 8 out of 10 samples studied, the short allele is the primary methylation target, whereas the long one is largely unmethylated (Figure 1C). In the case of AML7, both alleles were methylated, and in case C113 there was a mixture of unmethylated and methylated alleles with no difference in methylation. Furthermore, in C140 and AML5 many of the S alleles remain unmethylated; however this finding may well represent contamination from normal tissues in which both L and S alleles are largely unmethylated, or co-existence of unmethylated and methylated tumor cells. However, even in samples C140 and AML5, the number of methylated clones is higher for the S allele. Using methylation of >30% of CpG sites within the clone as an arbitrarily chosen cutoff for dense methylation, the short alleles were 3.1 times more methylated than the long alleles (p<0.001 by the Fisher exact test). Quantitatively similar results are obtained if the cutoff is 20%, 50%, or without using any cutoff (not shown). Taken together, these data demonstrate unequivocally preferential methylation of the short alleles even when they coexist with the long alleles within the same tumor.

We previously found that RIL methylation increases with age in normal colon [8]. To investigate whether this is influenced by the polymorphism, we studied 46 normal colon samples (17 homozygous short, 15 heterozygous and 14 homozygous long, obtained from individuals with comparable age) using 5 pyrosequencing methylation assays, each covering several CpG sites around the transcription start site, as well as exon 1 and intron 1 regions (Figure 1A, Table S1). We found that homozygous long samples were significantly less methylated compared to homozygous short in all the regions studied, both upstream and downstream of transcription start site (Figure 1D). Average methylation percent for the upstream region measured by assays A–F was 34.3±1.8 for homozygous short cases, 26.8±2.2 for heterozygous, and 19.4±1.1 for homozygous long cases and therefore the short alleles were 1.8 times more methylated in the normal colon. Finally, all samples showed increased methylation with age, independent of allele status (R = 0.42, p = 0.09 for homozygous short, R = 0.57, p = 0.03 for heterozygous, R = 0.69, p = 0.01 for homozygous long cases (Figure 1D).

**Figure 2:**
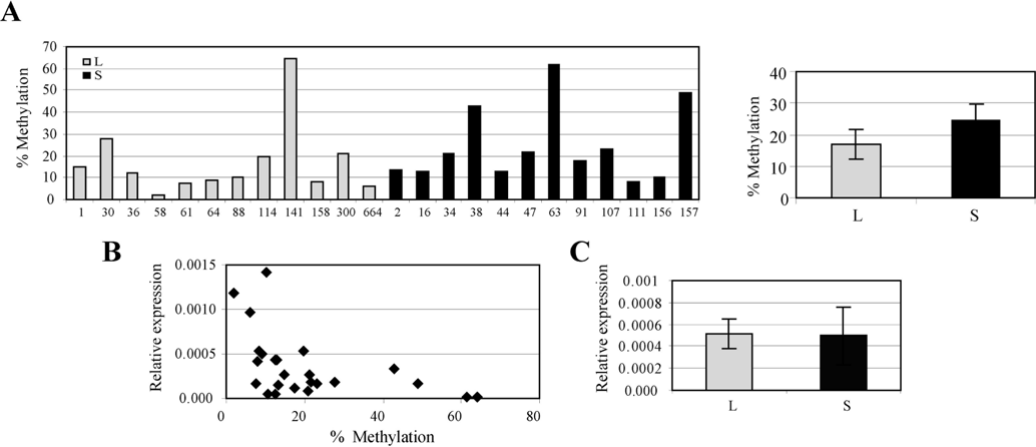
Allele-Specific Expression of RIL in Homozygous EBV-Transformed Cell Lines. (A) *Left*, Graphic representation of RIL methylation as determined by pyrosequencing with assay A in 12 long (L) and 12 short (S) EBV-transformed cell lines is shown (values represent averages of two independent pyro measurements for each sample). *Right*, average methylation values+/−SEM for homozygous long and homozygous short sample groups are shown. (B) Graphic representation of correlation between relative expression by quantitative PCR (RIL/GAPDH) and methylation for each EBV sample studied is shown. (C) Average expression values+/−SEM for unmethylated homozygous long (N = 8) and homozygous short (N = 5) cell lines with methylation below 15% are shown.

We were puzzled by the finding of differential allele methylation and thought that one likely explanation for such a difference is lower expression of the short alleles predisposing them to methylation. We therefore investigated whether the expression levels between the alleles are different *in vivo*. First, we obtained and genotyped 96 primary EBV-transformed lymphoblastoid cell lines established from normal individuals and found that 42 of those were homozygous short, 42 heterozygous, and 12 homozygous long. We then analyzed 24 cell lines (12 long and 12 short) for methylation status of RIL by pyrosequencing. We found variable levels of methylation among the cell lines, which was higher for short cell lines, although the difference was not statistically significant (Figure 2A). Nevertheless, this difference was quantitatively similar to what we saw in normal (non-malignant) colon tissues. We studied RIL expression in these samples by quantitative PCR and found it to correlate well with methylation (Figure 2B). We then excluded 11 cell lines with methylation above 15% and compared 8 unmethylated homozygous long with 5 unmethylated homozygous short cell lines and found no difference in expression (Figure 2C). This led us to conclude that there is no difference in expression between the long and short alleles in a physiologic setting.

**Figure 3:**
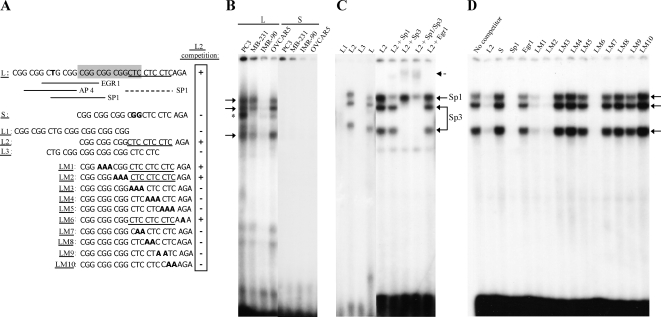
Identification of an Additional Sp1/Sp3 Binding Site in the Long Allele of RIL. (A) Sequences of EMSA probes. L probe sequence is shown on top. The 12-bp region absent in the short allele is shown in a grey box, and T, converted to G in the short allele, is shown in bold. Putative transcription factor binding sites (Transfac/MatInspector prediction) are shown. Mutations are shown in bold (probes LM1-LM10). Bolded nucleotides in the S probe correspond to the nucleotides different with the long probe. The CTCCTCCTC sequence is underlined. The ability of the probes to compete with the L2 probe is indicated on the right (+ or −). (B) Nuclear extracts from various cancer cells were incubated with the 33-bp L or 21-bp S probe. Note the presence of shifts with the L, but not with the S probe, which are indicated by arrows. (C) Nuclear extracts from MDA-MB-231 cells were incubated with three shorter 21-bp versions of the L probe (L1-L3). Notice binding only with the L2 probe, which has an intact 3CTC region. To identify the nature of proteins bound, supershift assays (arrows) of the L2 probe with specific antibodies for Sp1, Sp3, and Egr-1 were performed with MDA-MB-231 nuclear extract. (D) To confirm binding specificity of the L2 probe and to map the nucleotides involved in binding, competition experiments against the L2 probe were performed with 20× excess of unlabeled oligonucleotides using MDA-MB-231 cell line nuclear extract.

We next hypothesized that the insertion polymorphism creates binding sites for a protein that protects the long allele against methylation. To determine whether nuclear proteins can bind to the polymorphic region of the long or short alleles, we first performed electrophoretic mobility shift assays (EMSA) with nuclear extracts from different cell lines using double-stranded radiolabeled oligonucleotides that corresponded to bp −69 to −37 (L) or −57 to −37 (S) relative to the translation start site (see Figure 3A) of the long and short RIL alleles, respectively. We found strong DNA-binding activity for the L probe in 3/4 cell lines tested (PC3, MDA-MB-231, OVCAR5), whereas no detectable DNA binding activity was observed with the S probe using the same nuclear extracts, even at longer exposures (Figure 3B). Three major shifted bands were found as a result of this L probe DNA-binding activity. Specificity of those bands was confirmed by competition with unlabeled oligonucleotides (not shown). To map the region responsible for binding, we synthesized shorter versions of the L probe of an equal size to the original S probe: L1, corresponding to nucleotides −69 to −49; L2, nucleotides −57 to −37; and L3, nucleotides −63 to −43 (Figure 3A). EMSA results with the nuclear extracts from MDA-MB-231 cells indicated that, while probe L2 had a strong DNA-binding activity resulting in appearance of 3 distinct bands, probes L1 and L3 had no detectable DNA-binding activity (Figure 3C, first three lanes).

To identify the nature of proteins bound to the long allele, we performed supershift assays using different antibodies. When the L2 probe was mixed with nuclear extracts from MDA-MB-231, and then incubated with an anti-Sp1 antibody, the top band was diminished, and supershifted complexes were formed, indicating that the top band contained Sp1 (Figure 3C). Incubation of the L2 probe with the nuclear extracts in the presence of Sp3 antibody completely abolished the two lower bands and resulted in supershifted bands (Figure 3C). Incubation of the L probe with the nuclear extracts and both Sp1, Sp3 antibodies resulted in almost complete disappearance of all three bands, whereas an Egr-1 antibody had no effect on the shifts. We confirmed these results using recombinant human Sp1 (data not shown).

The results of EMSAs strongly suggested that binding of Sp1 and Sp3 proteins occurs in the 3CTC-containing region of the inserted region of the long allele. First, the L1 and L3 probes were unable to show any DNA-binding activity, and both do not have an intact 3CTC region. Second, the S probe which was also not bound by factors in the nuclear extracts, contains a 2CTC repeat, and in fact has only a 2-nucleotide difference with the L2 probe, thus being a naturally occurring mutant of L2 (Figure 3A). Finally, the 3CTC sequence has been previously shown to be a variant Sp1 site, important for regulation of several genes, including WT1 [9].

To map Sp1/Sp3 binding in this region, we generated a series of oligonucleotides with 3-bp mutations throughout the probe (LM1-LM6); and oligonucleotides with 2-bp mutations restricted to the 3CTC region (LM7-LM10) (see Figure 3A). We then competed the L2 probe with a 20-fold excess of various unlabeled competitors. These experiments revealed poor competition capacity of all the probes where the 3CTC repeat is absent (S, Egr1, LM3-LM5, LM7-LM10 probes), and efficient competition by all probes containing an intact 3CTC sequence (Figure 3D). Identical results were obtained in OVCAR-5 cells (data not shown).

**Figure 4:**
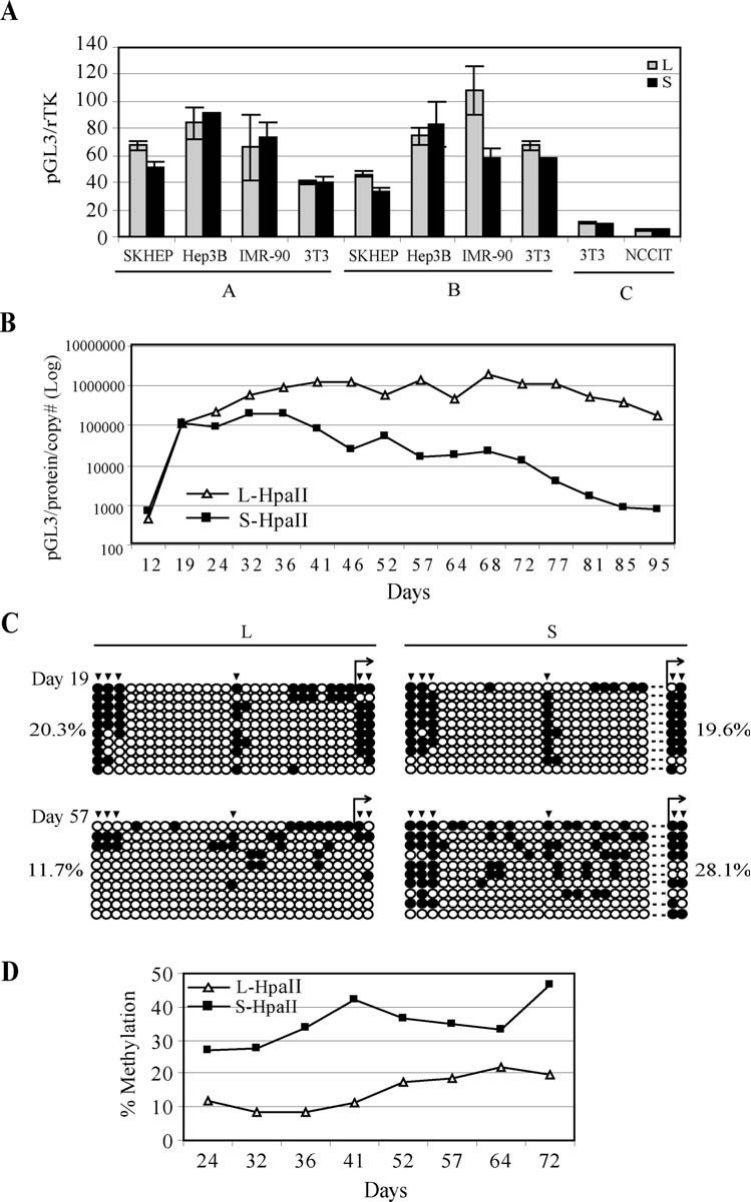
Induction of Hypermethylation and Silencing of the RIL Short Allele in Vitro. (A) Summary of transient allele-specific RIL/reporter gene expression in 4 cell lines. ∼0.2 KB (A), ∼0.6 KB (B) and ∼1.1 KB (C) RIL L and S promoter fragments were cloned upstream of pGL3 luciferase and transiently cotransfected with renilla thymidine kinase as a transfection control into the indicated cell lines in 6-well plates. Cells were harvested at 48 hrs and luciferase activities were analyzed. Luciferase/renilla ratios were plotted on the Y-axis. Transfections were performed in triplicates, and standard error bars for each experiment are shown on the graph. (B) Expression of stably transfected HpaII-seeded constructs. Long and short RIL allele-specific constructs (construct B, ∼0.6 KB) were seeded with HpaII methylase, stably cotransfected with pcDNA3.1 into NIH3T3 cells, selected in neomycin and pooled clones monitored for the indicated time period. Notice initial equal levels of expression, followed by maintenance of the long allele construct expression and rapid decline in expression of the short allele construct. (C) Bisulfite sequencing results for allele-specific seeded constructs. The methylation status of the transfected allele-specific constructs was analyzed by bisulfite sequencing at indicated time points, using plasmid-specific primers. Note equal methylation levels and presence of initial “seeds” of HpaII methylation (arrowheads) on day 19 and increased methylation spreading for the short allele construct at day 57. Percent methylation for each time point is indicated. (D) Pyrosequencing results for allele-specific seeded constructs. The methylation status of the transfected allele-specific constructs was analyzed by pyrosequencing at indicated time points, using plasmid-specific primers. One site was analyzed by the assay, which corresponds to CpG site # 9 in bisulfite sequencing figure. The pyrosequencing results shown agree with expression and bisulfite sequencing data.

Next, we sought to determine whether the additional Sp1/Sp3 binding site in the long allele affects transcription *in vitro*. We generated three different luciferase constructs driven by allele-specific RIL promoter fragments containing the polymorphic region of −588 to +19 (A), −217 to +19 (B) and −588 to +516 (C). In a series of transient transfections using 4 cell lines for constructs A and B and 2 cell lines for construct C, no significant differences in luciferase activity between the allele-specific constructs were observed (Figure 4A), suggesting that the additional Sp1 site had no substantial effect on RIL transcription. This was consistent with the earlier experiments in lymphocytes.

To determine whether the additional Sp1/Sp3 binding site confers protection against DNA methylation in vitro, we used the same constructs described earlier. We initially stably co-transfected unmethylated RIL allele-specific constructs (construct B) with neomycin-containing plasmids into the mammalian cell lines RKO and NIH3T3, and found that those maintained expression over 3 months, and were resistant to *de novo* methylation (data not shown), as previously reported for multiple genes [4]. Furthermore, it has been previously shown using GSTP1 gene as a model that aberrant methylation at a given promoter requires pre-existing “seeds” of methylation which trigger silencing [4]. To trigger silencing in our system, we used methylation “seeding” of allele-specific transgenes using HpaII methylase, which methylates 9.3% of the total CpG sites in construct B (13.5% of CpGs of the RIL promoter fragment), and co-transfected those with neomycin-containing plasmids into NIH3T3 cells. The plasmid methylation status prior to transfection was validated by HpaII methylation sensitive restriction enzyme digestion (Figure S3). After neomycin selection, pooled clones were passaged and analyzed for luciferase expression and methylation. We chose to use pooled clones to avoid the problem of insertion site variegation effect of single clones. Moreover, such a strategy is commonly accepted and has been successfully used before in a similar study [10]. We measured copy number for every time point and adjusted expression levels of each construct accordingly. As shown in Figure 4B, luciferase readings for both plasmids increased initially, concomitant with stable integration and neomycin selection. However, beyond day 36 post-transfection, RIL expression seemed to be stable for long alleles throughout the course of the experiment, but showed rapid declines for the short alleles. A completely independent seeding/transfection experiment with the same cell line showed similar results (Figure S4). We then analyzed methylation status of the constructs after stable transfection with bisulfite sequencing using transgene-specific primers (Figure 4C). We found that, at day 19 post-transfection, both constructs were equally methylated at HpaII sites with some evidence of spreading (average methylation 20.3% and 19.6% for long and short construct, respectively). However, at day 57, the RIL short allele construct remained methylated at the HpaII sites, and in addition, showed substantial evidence of methylation spreading (average methylation 28.1%). By contrast, the RIL long allele construct was found to have demethylation, at HpaII sites, which coexisted with modest methylation spreading on other sites and showed lower levels of methylation overall (average methylation 11.7%). Thus, the short allele construct was methylated 2.4-fold higher than the long allele construct on day 57 (P<0.001 by Fisher exact test). Interestingly, new methylation in the short construct did not seem to be spreading to CpG sites directly adjacent to HpaII-methylated sites, but was rather observed randomly between those sites, a phenomenon that has been reported previously [10]. Furthermore, to more fully characterize methylation status of the transgenes, we designed plasmid-specific pyrosequencing assays and measured several time points (Figure 4D). In general, our methylation and expression data agreed well. However, we must point out that the degree of loss of expression observed for the short alleles cannot be fully explained by methylation gains, thus factors other than methylation (i.e. preceding chromatin changes) might have contributed to the decline in luciferase activity, with methylation following this. In summary, the stably integrated RIL long allele construct is protected from methylation and time-dependent silencing, consistent with several previous observations that Sp1 sites confer protection against DNA methylation [11].

## Discussion

Here we have identified a genetic variation in the promoter region of RIL that results from a presumed insertion of 12 bp around transcription start. This naturally occurring insertion results in a significant 1.8-fold lower methylation of the gene in normal samples, and a 2.1-fold to 3.1-fold lower methylation in cancer that we have traced to the creation of a new Sp1/Sp3 binding site. This finding is consistent with previous reports identifying Sp1 sites as regulatory DNA elements protecting CpG islands from methylation during embryogenesis [12]; [13] and can now be extended to hypermethylation in aging and cancer. This model is consistent with the concept proposed by Turker and others [14], which suggests that methylation is a non-random process that results in a misbalance between DNA methylation-promoting events (e.g. abnormal spreading of methylation in *cis* from adjacent “methylation centers”, repeat elements; and methylation “seeding”) and DNA methylation preventing events (i.e., Sp1 elements in the promoters as well as other insulator sequences, and proteins bound to those sequences; as well as demethylation). Our study demonstrates that trans-acting factors can affect hypermethylation in aging and cancer, and establish a new concept in gene inactivation whereby genetic polymorphisms in protein binding sites result in variable susceptibility to epigenetic silencing. A relationship between genetic polymorphisms and aberrant methylation was recently described for MSH2 and was named heritable germline epimutation [15]. A similar relationship has also been described for MGMT [16]. In these cases, the exact mechanism of such predispositions is unknown. It is attractive to think that these heritable epimutations mechanistically represent polymorphisms that influence DNA-binding proteins, as in the case of RIL. It would now be of interest to determine how many genes hypermethylated in cancer are affected by such promoter polymorphisms.

The mechanism of Sp1/Sp3 protection against silencing remains unclear. Our studies of RIL expression both *in vivo* in EBV-transformed cells (Figure 2) and in vitro (Figure 4A), using allele-specific reporter gene assays showed no difference in expression levels between the two alleles of RIL. Rather, our results with the in vitro seeding model are generally consistent with the idea that it protects against time-dependent silencing. However, the degree of methylation spreading observed in the short alleles cannot quantitatively explain the observed prominent drop in transcription over time (Figure 4B–D), and we suggest that the effects of the additional Sp1/Sp3 site are to create local protection against a repressive chromatin environment (e.g. histone based silencing) which, secondarily, leads to the observed DNA methylation differences. There are also clear limitations to short term in-vitro assays in modeling in-vivo situations. The experiments described attempt to reproduce in a relatively short period of time an in-vivo situation which evolves over decades of human aging. Nevertheless, it provides an experimental validation of the in-vivo situation, and shows that the short alleles are more prone to time dependent silencing.

Our findings are in line with previous *in-vitro* studies of the APRT gene that suggested that different Sp1 sites might have different functions; some involved in regulation of gene expression, whereas others in protection against DNA methylation [11]. In support of this model, recent data pointed out that Sp1 may have boundary activities and prevent heterochromatin spreading in yeast, which lack Sp1 homologues: targeting of Sp1 to transgenes in yeast cells unexpectedly revealed barrier activity, which was independent of a transactivation domain [17]. It is tempting to speculate that the Sp1/Sp3 site created by the polymorphism in RIL plays such a role.

Identification of allelic variants of genes that have different susceptibility to methylation in aging and cancer is an important new link between human genetic variation and epigenetic silencing. In this study, we propose a plausible mechanism responsible for this unique biological phenomenon.

## Materials and Methods

### Cell Lines and Culture Conditions

HCT116, RKO and NIH3T3 cells were grown in high glucose DMEM (Life Technologies, Gaithersburg, MD) plus 10% fetal bovine serum (FBS; Intergen, Purchase, NY); OVCAR-5, PC3, MB-231 and the EBV-transformed cells were grown in RPMI 1640 media plus 10% FBS. All cells were grown in plastic tissue culture plates in a humidified atmosphere containing 5% CO_2_ at 37°C.

### Human Samples

Samples of primary colon cancers and primary leukemias were obtained from established tissue banks at M. D. Anderson Cancer Center and Johns Hopkins University. Of 44 primary colon cancer samples, 10 were stage II, 12 stage II, 7 stage IV, and 15 were of unknown stage. EBV-transformed lymphocyte cell lines established from normal Caucasian individuals were obtained from Baylor College of Medicine. All samples were collected from consenting patients according to institutional guidelines.

### DNA, RNA extraction, and cDNA Synthesis

DNA was extracted by using conventional phenol-chloroform method. Total cellular RNA was extracted with TriZOL (Invitrogen, Carlsbad, CA) according to the manufacturer protocol and resuspended in DEPC-treated water. Reverse transcription reactions were performed using MMLV-RT (Roche, Indianapolis, IN) on 2 µg of total RNA per reaction according to the manufacturer's protocol.

### Genotyping

Genotyping to determine RIL allele status was performed by genomic PCR using del7 (TCCAGGCGCACAGGGAGC) and del8 (GCCTGAGCCGGACTCTGAGGA) primers. PCR products were separated on 6% polyacrylamide gel and were classified as short, long, or heterozygous, depending on the size and number of bands. Several cases were verified by direct sequencing.

### Bisulfite Modification of DNA

Bisulfite induces deamination of unmethylated cytosines, converting unmethylated CpG sites to UpG without modifying methylated sites. Bisulfite treatment of genomic DNA was performed as described [18]. DNA was extracted using standard phenol-chloroform method. After extraction, 2 µg of DNA were used for bisulfite treatment. DNA was denatured in 0.2 N NaOH at 37°C for 10 min and incubated with 3 M sodium bisulfite at 50°C for 16 h. Bisulfite-converted DNA was purified using the Wizard cleanup system (Promega, Madison, WI) and desulfonated with 0.3 N NaOH at 25°C for 5 min. DNA was then precipitated with ammonium acetate and ethanol, washed with 70% ethanol, dried and resuspended in H_2_O.

### COBRA Analysis and Bisulfite Sequencing

PCR reactions were carried in 50-µl reactions using the COBRA primers (forward, GTTTATTAGGYGGAAGTTTTAGG and reverse, AACCAATCCAAACRCACAA) are complementary to the RIL antisense strand. In each reaction, 2 µl of bisulfite-treated DNA were used, as well as 1.25 mM deoxynucleotide triphosphate, 67 mM Tris-HCl, pH = 8.8, 16 mM ammonium sulfate, 10 mM β-mercaptoethanol, 0.1 mg/ml bovine serum albumin, 10 pmol of primers and 1 unit of Taq polymerase. All PCR reactions were performed using a hot start at 95°C for 5 min. After amplification PCR products were digested with the HpyCH4IV restriction enzyme (New England Biolabs, Ipswitch, MA), which digests alleles that were methylated prior to bisulfite treatment. The digested DNA was separated in nondenaturing polyacrylamide gels and stained with ethidium bromide. The proportion of methylated *versus* unmethylated product (digested *versus* undigested) was quantitated by densitometric analysis, performed using a Bio-Rad Geldoc 2000 digital analyzer equipped with the Quantity One version 4.0.3 software. In case of bisulfite sequencing, restriction enzyme digestion step was omitted, PCR products were directly cloned into a TOPO-TA vector (Invitrogen, Carlsbad, CA) and individual clones were sequenced.

### Pyrosequencing

To study methylation in normal colon, primary cancer samples and in 3T3 cells transfected with HpaII-seeded constructs, we used the pyrosequencing method [19]. For PCR, we used 2 µL bisulfite treated DNA, 1.25 mM deoxynucleotide triphosphate, 1 unit of Taq polymerase and the PCR buffer mentioned above, 10 pmol forward primer, 1 pmol reverse-universal primer, and 9 pmol universal biotinylated primer (assays E, F, G). In assay A, we used 1 pmol forward-universal primer, 10 pmol reverse primer and 9 pmol universal biotinylated primer. In assay B, the reverse primer was directly biotinylated. In plasmid-specific assays, we used a two-step PCR approach using nested primers, and in the second step, we used 10 pmol forward primer, 1 pmol reverse-universal primer, 1 pmol reverse primer and 9 pmol universal biotinylated primer. All primer sequences and conditions are shown in Supplementary Table S1. The final biotin-labeled PCR product was captured by Streptavidin Sepharose HP (Amersham Biosciences, Sweden). PCR products bound on the bead were purified and made single stranded using a Pyrosequencing Vacuum Prep Tool (Biotage, Sweden). The sequencing primers (0.3 µmol/L; Supplementary Table S1) were annealed to the single-stranded PCR product, and pyrosequencing was done using the PSQ HS 96 Pyrosequencing System (Biotage, Sweden). Quantification of cytosine methylation was done using the provided software (PSQ HS96A 1.2).

### Nuclear Cell Extracts

Nuclear extracts were prepared with the NE-PER nuclear extraction reagents (Pierce, Rockford, IL), according to manufacturer's instructions. Briefly, cells were lysed in hypotonic CER buffer with added protease inhibitors and the cytoplasmic fraction was separated by centrifugation. The nuclear pellet was resuspended in hypertonic NER buffer with added protease inhibitors and incubated on ice for 40′, vortexed every 10′. For nuclear extracts, the soluble proteins in the lysate were separated by centrifugation. The protein content was quantified using the Bio-Rad protein assay.

### Electrophoretic Mobility Shift Assay

All EMSA probes were generated by annealing synthetic complementary oligonucleotides (Invitrogen) corresponding to the deletion/insertion region of the RIL promoter, sequences are shown in Figure 4A. ^32^P-end-labeled oligonucleotides (100,000 cpm) were incubated for 30 min on ice with 5–10 µg of nuclear extract or, alternatively, with 200 ng of recombinant human Sp1 (Promega) in 15 µl of binding buffer containing 10 mM Tris-HCl, pH 7.5, 0.5 mM EDTA, 0.5 mM dithiothreitol, 4% glycerol, 1 mM MgCl_2_, 50 mM NaCl, and 1 µg of poly(dI-dC). The binding reaction was incubated on ice for 30 min. Competition reactions were performed with a 20-fold molar excess of unlabeled double-stranded competitor DNA. For supershift analysis, the nuclear extracts were incubated with polyclonal anti-human Sp1, Sp3, Egr-1 and AP-2 antibodies (Santa Cruz Biotechnology, Santa Cruz, CA) for 1 h following the binding reaction with labeled probe. The DNA-protein complexes were separated on a 5% native polyacrylamide gel in 0.5× TBE buffer for 14 h at +4°C at 90 V.

### Construction and Transfection of RIL Allele Specific Luciferase Reporter Plasmids

DNA from human colon cancer cells HCT116 and RKO was extracted as previously described, using phenol chloroform methods. The more 3′ located transcription start site (Figure 1A) was arbitrarily chosen as +1. The 236-bp genomic fragment from HCT116 cells (homozygous long) and the 224-bp genomic fragment from RKO cells (homozygous short) spanning from −217 to +19 (construct A) were generated by PCR from genomic DNA using RIL-LUC1 (CGGAGCTCTCTCTGAGAGCTGAGTGGGG) and RIL-LUC2 (GCAAGCTTCTGAGCCGGACTCTGAGG) primers. The 607-bp genomic fragment from HCT116 cells and the 595-bp genomic fragment from RKO cells spanning from −588 to +19 (construct B) were generated by PCR from genomic DNA using RIL-LUC3 and RIL-LUC2 primers. Both RIL-LUC1 and RIL-LUC3 (GGGAGCTCCCTTACTGGCCTCCACAAAC) primers contained SacI restriction enzyme sites, whereas RIL-LUC2 primer contained HindIII restriction site. The genomic fragments from HCT116 cells (homozygous long) and from RKO cells (homozygous short) spanning from −588 to +516 (construct C) were generated by PCR from genomic DNA using RIL-LUC4 (GTGCTAGCCCTTACTGGCCTCCACAAAC) and RIL-LUC5 (CCAAGCTTGGACCTGCGAGCAGACAAGCCTCATTTTGCCCCAGATCTTC) primers. In construct C, the reverse primer contained additional intronic sequence to assure correct splicing of the exon 1. The PCR products were digested with NheI and HindIII enzymes (NEB) and subcloned in the promoterless pGL3 basic vector (Promega) and, generating the allele-specific constructs A (236/224-bp), B (607/595-bp) and C (1104/1092-bp). The constructs were confirmed by DNA sequencing. Plasmids were transfected using Lipofectamine2000 (Invitrogen), according to manufacturer's protocol.

### Transient Transfections and Luciferase Assay

The cells were grown in 6-well plates to 60% confluence for at least 18 h and then were transiently transfected using Fugene6 reagent (Roche, Indianapolis, IN) with 1 µg of a firefly luciferase reporter gene and 50 ng of the *Renilla* luciferase reporter gene, driven by the thymidine kinase promoter (Promega). Cotransfection was performed by adding 1 µg of expression constructs to the DNA solutions. Luciferase activity was determined using the dual luciferase reporter assay system (Promega) in a microplate Monolight 3010 luminometer (BD Biosciences, San Diego, CA) according to the manufacturer's instructions. Normalization of transfection efficiency was based on cotransfected thymidine kinase *Renilla* luciferase activities.

### HpaII Methylation Seeding, Stable Transfections, Bisulfite PCR, and Pyrosequencing

RIL allele-specific constructs (construct B, ∼0.6 KB) were methylated using HpaII methylase (NEB) according to manufacturer's protocol. Methylation was confirmed by resistance to restriction to HpaII enzyme (Figure S3). For stable transfections, allele-specific luciferase constructs were co-transfected with pcDNA3.1 neomycin-containing vector in 20∶1 ratio into 3T3 cells using Lipofectamine 2000 (Invitrogen). Methylation analysis of transfected constructs was done by amplification of bisulfite treated DNA, using plasmid-specific primers (F: GAGAGTTGAGTGGGGGTGT, R: TTCCATAATAACTTTACCAACAATACC) followed by bisulfite sequencing of single clones. For pyrosequencing, these primers were used for the 1^st^ step PCR, followed by second step with nested primers (Supplementary Table S1). One site was analyzed by the assay, which corresponds to CpG site # 9 in bisulfite sequencing (Figure 4C).

### Real-Time PCR

Q-PCR was used to quantify RIL mRNA in total RNA isolated from EBV-transformed lymphoblastoid cell lines and reverse transcribed into cDNA, and also to estimate relative plasmid copy numbers in the genomic DNA isolated from pooled transfected NIH3T3 cells. We used TaqMan Universal PCR Master Mix and ABI Prism 7000 Sequence Detection System (Applied Biosystems, Foster City, CA). The primers and TaqMan probes were designed by using Primer Express software (Applied Biosystems), except for RIL expression assay which was custom-designed and purchased from Applied Biosystems (cat. No. Hs00184792_m1). All probes were labeled with the 6-carboxyfluorescein fluorophore (6-FAM) and a nonfluorescent MGB quencher. To normalize RIL expression, GAPDH was used as reference standards; to estimate the relative plasmid copy number per cell, murine beta-globin was used as endogenous reference. Primer and probe sequences used in the experiments were as follows: (1) RIL assay, custom-designed and purchased from Applied Biosystems, cat. No. Hs00184792_m1; (2) GAPDH assay, gapdh-284F, 5′-ATGGAAATCCCATCACCATCTT-3′; gapdh-340R, 5′-CGCCCCACTTGATTTTGG-3′; gapdh-307T (MGB probe, FAM fluorophore labeled), 5-CGCAGTTGGGCACTT-3; (3) Luciferase assay, luc-161F, 5′-CACATATCGAGGTGGACATC-3′; luc-220R, 5′- GCCAACCGAACGGACATTT-3′; luc-184T (MGB probe, FAM fluorophore labeled), 5-CTTACGCTGAGTACTTC-3; (4) Murine beta-globin assay, Mu-bglo-239F, 5′- AGGCCCATGGCAAGAAAGT-3′, Mu-bglo-306R, 5′-GCCCTTGAGGCTGTCCAA-3′, Mu-bglo-259T (MGB probe, FAM fluorophore labeled), 5-ATAACTGCCTTTAACGATG-3. All FAM probes were custom synthesized by Applied Biosystems. The primers were used at 900 nM and the probes at 100 nM concentrations. We used the RNA or DNA amount giving the linear range of response, typically CT range of 20–30 amplification cycles. We amplified each gene in separate reactions in triplicates, using a protocol recommended by manufacturer. PCR without reverse transcriptase was performed for each sample to control for the possible interference from gDNA contamination. The threshold amplification cycles (CT) at the normalized reporter signal minus the baseline signal level of 0.2 for RIL, GAPDH, Luciferase and murine beta-globin were determined and their differences deltaCT(GAPDH-RIL) and deltaCT(murine beta globin-Luciferase) were calculated.

### Statistics

Statistical differences of bisulfite sequencing data were calculated using Fisher exact test. To analyze methylation levels across the CpG island, we calculated average±standard error of the mean for each region studied, and performed T-test using Microsoft Excel. To estimate correlation between average methylation density (regions A–F) and age for each region in normal colon samples, non-parametric two-tail Spearman test (95% confidence interval) was used (Graphpad Prizm 4 software). All EMSA experiments were done in triplicates and yielded identical results. Luciferase assays on cells were performed three separate times, and the results were expressed as average±standard error of the mean calculated using Microsoft Excel.

## Supporting Information

Figure S1 Differential methylation of RIL alleles in homozygous and heterozygous tumors revealed by COBRA assay. Here, a RIL COBRA gel image for representative colon cancer and paired normal samples, as well as ML-1 cell line is shown. Allele status of samples is indicated on the top of the gel. In the middle of the gel, the upper arrow indicates an allele-specific band, which is seen only if the long allele undergoes methylation, whereas the lower arrow points to the allele-specific band that is seen if the short allele is methylated. In the bottom of the gel, an allele non-specific band is seen, which is always present if RIL is methylated, regardless of allele status. After digestion of the COBRA products obtained from several homozygous and heterozygous tumors, we observed that among the 2 middle allele-specific bands, the predominant one is the lower band, which represents methylated short allele molecules. Note methylation of only the S allele in heterozygous cases C110 and C140. These findings led us to a suspicion that the short allele is more methylated than the long.
(0.27 MB TIF)

Figure S2 Mixing study for pyrosequencing assay A. Normal blood (unmethylated) DNA was mixed with methylated DNA (RKO) at known ratios and subjected to bisulfite treatment. Then, pyrosequencing assay A (Figure 1A) was tested for bias of estimating methylation by studying mixed RKO, which has near 100% methylation as determined previously by COBRA, bisulfite sequencing, and pyrosequencing assays. The graph shows pyrosequencing measurement values determined for bisulfite-treated DNA of mixed RKO and normal blood in the ratio shown on the X-axis. Measured values are plotted alongside with expected methylation values, which are in fact very similar. Thus, we conclude that there is no bias in amplifying unmethylated vs. methylated DNA with the assay.
(0.04 MB TIF)

Figure S3 The HpaII seeding of allele-specific construct B was done by HpaII methylase treatment and the plasmid methylation status prior to transfections was validated first by HpaII restriction enzyme (methylation sensitive) digestion. The gel image shows that while unmethylated allele-specific plasmids (lane 2, RIL-long and lane 4, RIL-short constructs) were digested into low size fragments, seeded plasmid were resistant to digestion (lane 1, RIL-long and lane 3, RIL-short methylation seeded constructs). λM1, HindIII marker and M2, 100-bp marker.
(0.70 MB TIF)

Figure S4 Expression of stably transfected HpaII-seeded constructs in an independent second experiment. Long and short RIL allele-specific constructs (construct B, ∼0.6 KB) were seeded with HpaII methylase, stably cotransfected with pcDNA3.1 into NIH3T3 cells, selected in neomycin and pooled clones monitored for the indicated time period. Notice initial equal levels of expression (Day 18), followed by increase or maintenance of the long allele construct expression and in contrast, declining expression of the short allele construct.
(0.06 MB TIF)

Table S1 Primer Sequences for the Genes Studied, PCR Conditions, and Assay Locations.
(0.02 MB PDF)
