# Supplementary figures and images for: An Sp1/Sp3 Binding Polymorphism Confers Methylation Protection
Source: PLoS Genet. 2008 Aug 22;4(8):e1000162. doi: 10.1371/journal.pgen.1000162 (PMC2515197; doi:10.1371/journal.pgen.1000162)

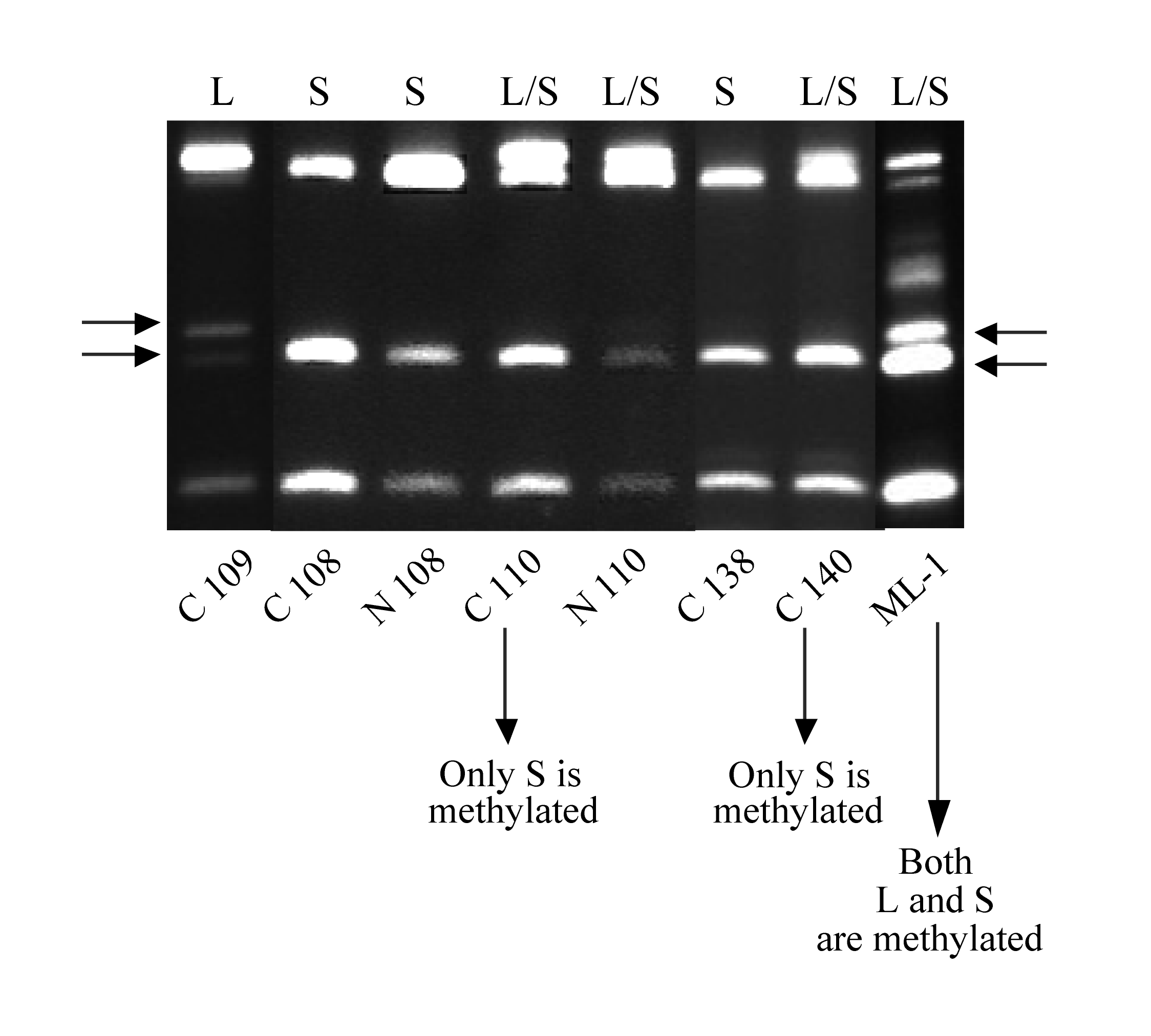

Supplement: Figure S1 — Differential methylation of RIL alleles in homozygous and heterozygous tumors revealed by COBRA assay. Here, a RIL COBRA gel image for representative colon cancer and paired normal samples, as well as ML-1 cell line is shown. Allele status of samples is indicated on the top of the gel. In the middle of the gel, the upper arrow indicates an allele-specific band, which is seen only if the long allele undergoes methylation, whereas the lower arrow points to the allele-specific band that is seen if the short allele is methylated. In the bottom of the gel, an allele non-specific band is seen, which is always present if RIL is methylated, regardless of allele status. After digestion of the COBRA products obtained from several homozygous and heterozygous tumors, we observed that among the 2 middle allele-specific bands, the predominant one is the lower band, which represents methylated short allele molecules. Note methylation of only the S allele in heterozygous cases C110 and C140. These findings led us to a suspicion that the short allele is more methylated than the long. (0.27 MB TIF) [file pgen.1000162.s001.tif]

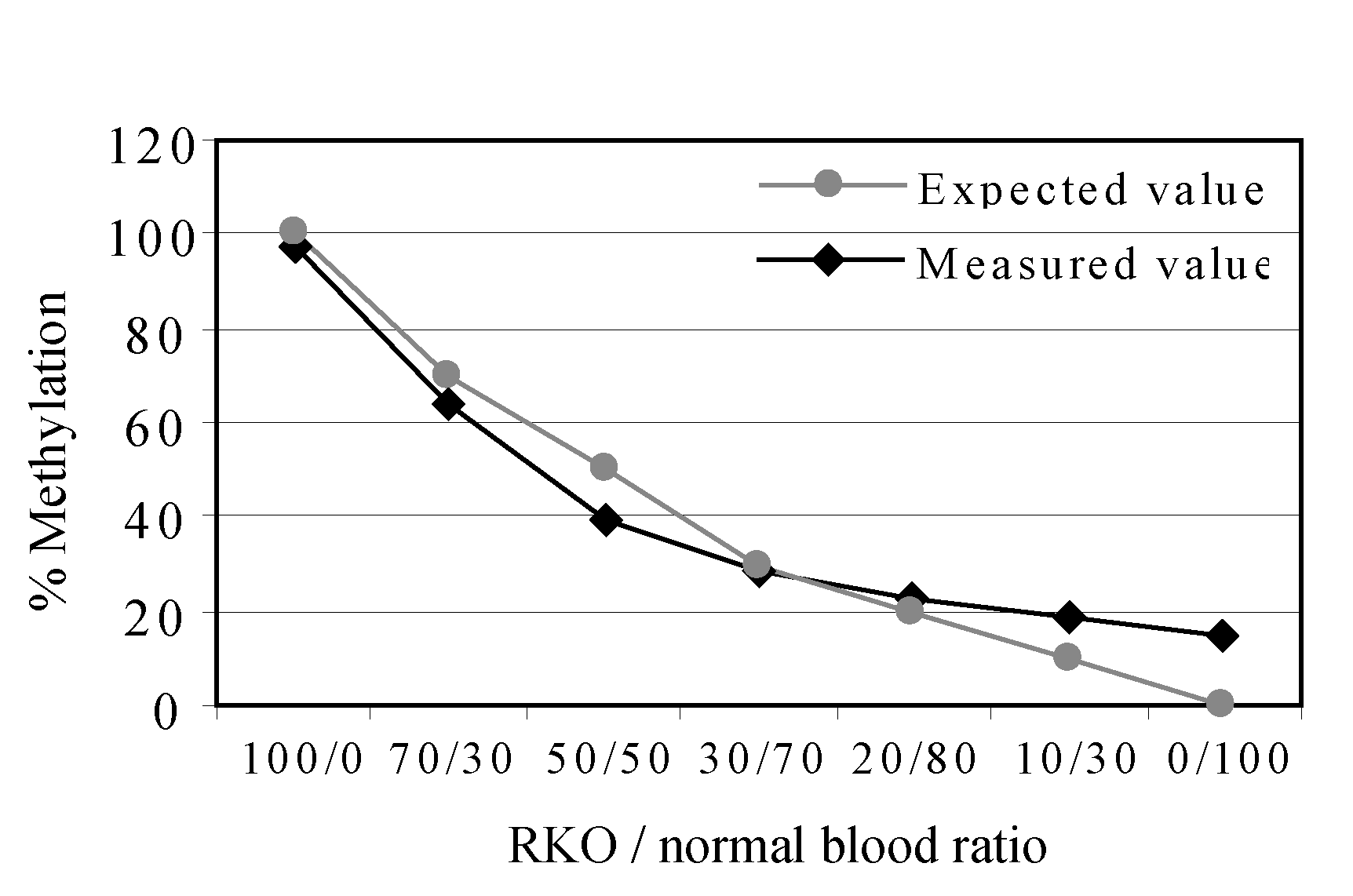

Supplement: Figure S2 — Mixing study for pyrosequencing assay A. Normal blood (unmethylated) DNA was mixed with methylated DNA (RKO) at known ratios and subjected to bisulfite treatment. Then, pyrosequencing assay A (Figure 1A) was tested for bias of estimating methylation by studying mixed RKO, which has near 100% methylation as determined previously by COBRA, bisulfite sequencing, and pyrosequencing assays. The graph shows pyrosequencing measurement values determined for bisulfite-treated DNA of mixed RKO and normal blood in the ratio shown on the X-axis. Measured values are plotted alongside with expected methylation values, which are in fact very similar. Thus, we conclude that there is no bias in amplifying unmethylated vs. methylated DNA with the assay. (0.04 MB TIF) [file pgen.1000162.s002.tif]

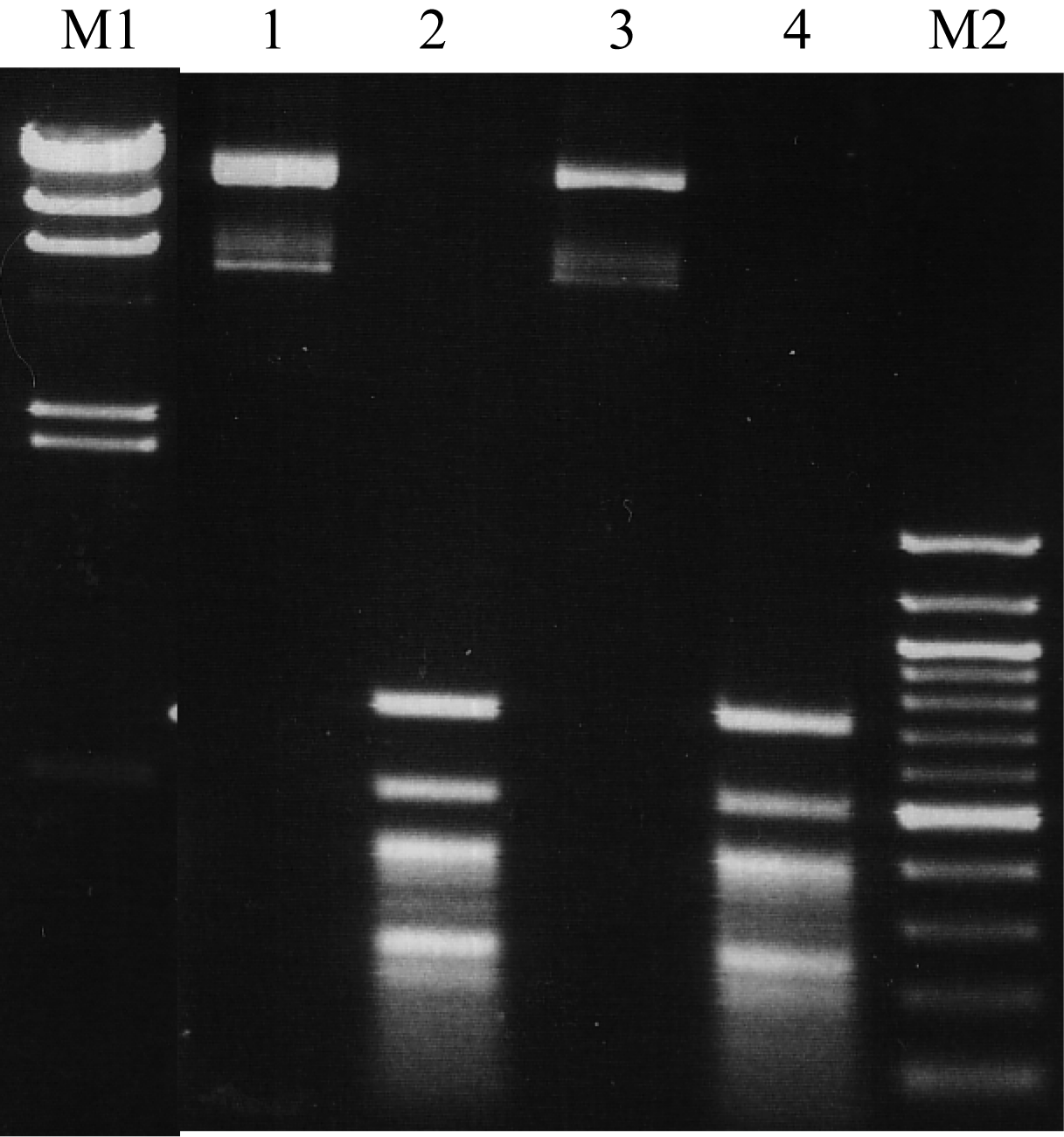

Supplement: Figure S3 — The HpaII seeding of allele-specific construct B was done by HpaII methylase treatment and the plasmid methylation status prior to transfections was validated first by HpaII restriction enzyme (methylation sensitive) digestion. The gel image shows that while unmethylated allele-specific plasmids (lane 2, RIL-long and lane 4, RIL-short constructs) were digested into low size fragments, seeded plasmid were resistant to digestion (lane 1, RIL-long and lane 3, RIL-short methylation seeded constructs). λM1, HindIII marker and M2, 100-bp marker. (0.70 MB TIF) [file pgen.1000162.s003.tif]

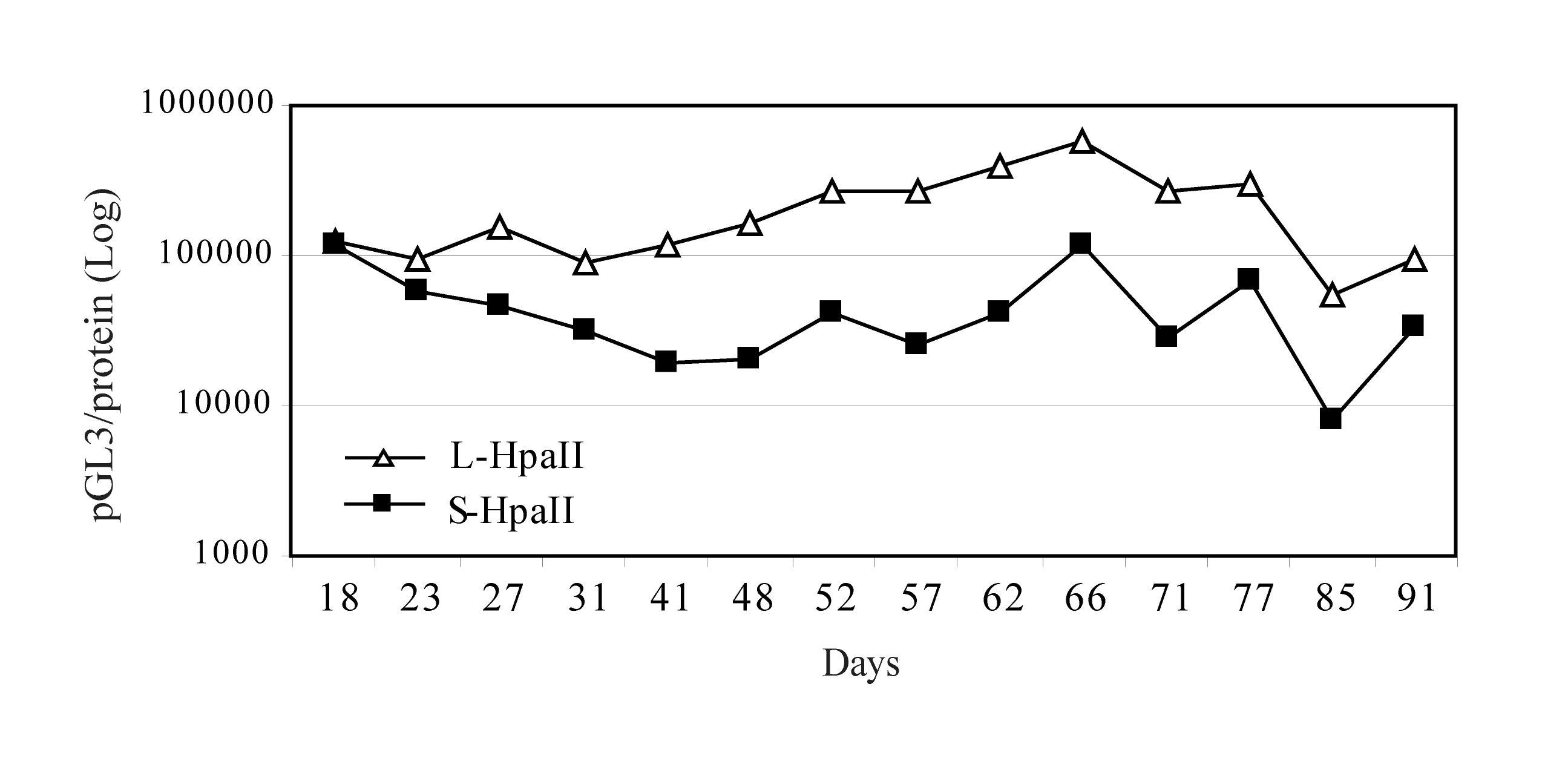

Supplement: Figure S4 — Expression of stably transfected HpaII-seeded constructs in an independent second experiment. Long and short RIL allele-specific constructs (construct B, ∼0.6 KB) were seeded with HpaII methylase, stably cotransfected with pcDNA3.1 into NIH3T3 cells, selected in neomycin and pooled clones monitored for the indicated time period. Notice initial equal levels of expression (Day 18), followed by increase or maintenance of the long allele construct expression and in contrast, declining expression of the short allele construct. (0.06 MB TIF) [file pgen.1000162.s004.tif]
