# Supplementary material for: An Sp1/Sp3 Binding Polymorphism Confers Methylation Protection
Source: PLoS Genet. 2008 Aug 22;4(8):e1000162. doi: 10.1371/journal.pgen.1000162 (PMC2515197; doi:10.1371/journal.pgen.1000162)
Supplement: Table S1 — Primer Sequences for the Genes Studied, PCR Conditions, and Assay Locations. (0.02 MB PDF) [file pgen.1000162.s005.pdf]

Supplementary Table S1: Primer Sequences for the Genes Studied, PCR Conditions, and Assay Locations.
F: Forward primer, R: Reverse primer, U-Biotin-universal primer, S: sequencing primer

Name	Primer sequence	Location relative to TSS and number of CpGs analyzed	Annealing temp. (PCR cycle no.)
Pyrosequencing Assay A	F- GGGACACCGCTGATCGTTTAGGAGGT ATTTGAGTTGGGGTA<br>R- CCCACTCAACTCTCAAAAAT<br>U- GGGACACCGCTGATCGTTTA<br>S- TACTCAACCTTAAATCTACA	-276 to –306 (4 sites)	51(48)
Pyrosequencing Assay B	F- TTTGTGAGTTTGGATTGGT<br>R- CCCCAAATAAACCCTCCAT<br>S- TTTATTTAGTTTTTAGAGAT	-223 to –242 (6 sites)	51(48)
Pyrosequencing Assay E	F- GATATATAGGGGATTTGGGTTT<br>R- GGGACACCGCTGATCGTTTATCCTCA AAATCCRACTCAAACT<br>U- GGGACACCGCTGATCGTTTA<br>S- AGTTTTAGGGAGAAGGTT	+57 to +69 (3 sites)	51(48)
Pyrosequencing Assay F	F- GATATATAGGGGATTTGGGTTT<br>R- GGGACACCGCTGATCGTTTATCCTCA AAATCCRACTCAAACT<br>U- GGGACACCGCTGATCGTTTA<br>S- GAGAGAATTAGGTTTTAGATATG	+149 to +168 (4 sites)	51(48)
Pyrosequencing Assay G	F- GAGTTTTTGGGTTTTTGTAGAG<br>R- GGGACACCGCTGATCGTTTA TCCCACCTAAACCTATCTACC<br>U- GGGACACCGCTGATCGTTTA<br>S- GGTTTTTTTAGATGAGGTTA	+370 to +384 (3 sites)	55(42)
